# Formation of self-organized Zircaloy-4 oxide nanotubes in organic viscous electrolyte via anodization

**DOI:** 10.1186/1556-276X-9-553

**Published:** 2014-10-04

**Authors:** Ghafar Ali, Yang Jeong Park, Hyun Jin Kim, Sung Oh Cho

**Affiliations:** 1 Department of Nuclear and Quantum Engineering (NQE), Korea Advanced Institute of Science and Technology (KAIST), 373-1 Guseong, Yuseong, Daejeon 305-701, Republic of Korea; 2 Nanomaterials Research Group, Physics Division, PINSTECH, Islamabad, Pakistan

**Keywords:** Anodization, Zircaloy, Nanotubes, SEM, TEM, XPS

## Abstract

This work reports the formation of self-organized Zircaloy-4 (Zr-4) oxide nanotubes in viscous organic ethylene glycol (EG) electrolyte containing a small amount of fluoride salt and deionized (DI) water via an electrochemical anodization. The structure, morphology, and composition of the Zr-4 oxide nanotubes were studied using X-ray diffraction (XRD), scanning electron microscope (SEM), transmission electron microscope (TEM), EDX, and XPS. SEM results showed that the length of the nanotubes is approximately 13 μm, and TEM results showed that the inner diameter of the Zr-4 oxide nanotubes is approximately 20 nm with average wall thickness of approximately 7 nm. XRD and selected area electron diffraction pattern (SAED) results confirmed that the as-anodized Zr-4 oxide nanotubes have cubic crystalline structure. Both cubic and monoclinic phases were found after annealing of Zr-4 oxide nanotubes. The tubular structure morphology of Zr-4 oxide nanotubes did not remain intact after annealing which is attributed to the elimination of F species from the annealed nanotubes.

## Background

Zirconium alloys (Zircaloy-2 and Zircaloy-4) are important alloys that are widely used as cladding materials for fuel rods in the light water nuclear reactors due to their low-neutron absorption cross section, high corrosion resistance, high ductility, and adequate hardness during normal operating conditions [1-3]. It is well known that nanostructured materials possess excellent properties compared to their bulk counterparts because of their high surface-to-volume ratio. The surface-to-volume ratio increases when the size of the materials decreases down to nanometer scale. The enhancement in the critical heat flux (CHF) has been reported when Zr-4 oxide nanostructures were implied in a pool-boiling experiment compared to the bulk materials as a result of good surface wettability [4,5]. The nanostructures exhibit superhydrophilic properties, and, thus, heat transfer coefficient and accordingly CHF can be much increased. Similar increase in the electrochemical corrosion resistance (approximately 100 times) has been observed in the anodic Zr-4 oxide compared to the as-received one [6]. Nanostructures of different morphologies can be produced on the surface of zircaloy using a simple, versatile, and cost-effective technique of anodization. The morphology, pore diameter, and length of the nanostructure can be easily tailored by tuning the anodization parameters. Anodization is so far applied to various metals such as Al [7-9], Ti [10,11], Ta [12], Hf [13], Nb [14], and alloys [15]. Depending upon the anodizing conditions, nanotubular or nanoporous structures can be selectively produced on the surface of materials as a result of anodization.

Many articles about the formation of zirconium oxide (ZrO_2_) nanotubes have reported by anodization of Zr foil in various electrolytes [16-23]; however, very little attention is given to zircaloy oxide nanotubes to date. Earlier data indicates that very limited reports have been available about the oxide layer formation on the surface of Zr-2 [24-26] and Zr-4 [6] via anodization in various aqueous electrolytes. Most of the anodic oxide layers formed on zircaloy were mainly composed of compact and barrier-type structure with limited thickness; however, the formation of some irregular micro- and nanopores has also been reported in Zr-2 oxide layer [25]. Kim et al. recently reported the formation of Zr-4 oxide nanotubes on the surface of Zr-4 in a HF acid-based aqueous electrolyte using anodization [4,5]. It is known that HF is highly toxic, extremely corrosive, and hazardous. It always needs fume hood and special tools to handle it. In addition, HF-based electrolyte always produced nanotubes with very short length due to severe dissolution [27] which may not be useful in certain applications.

Here in this article, we used an environmentally benign organic viscous solvent for the synthesis of Zr-4 oxide nanotubes. Zr-4 oxide nanotubes have been prepared in ethylene glycol (EG) electrolyte containing fluoride salt (NH_4_F) and deionized (DI) water. Electrolytes based on organic solvents such as EG and glycerol are highly ideal for growing very long Zr-4 oxide nanotubes like TiO_2_ nanotubes as reported in our previous work [10,11]. The fabrication of high aspect ratio Zr-4 oxide nanotubes on the surface of Zr-4 may be useful for many applications including CHF. Further work on the anodic oxidation of Zr-4 in different organic-based electrolytes is in progress for tailoring various aspects of the oxide layer like adhesion and morphology required in some specific applications which will be reported in future publications.

## Methods

The as-received Zr-4 sheets were cut into 20 × 25 × 0.7 mm^3^ and cleaned by sonicating in acetone, isopropyl alcohol, and methanol without mechanical, chemical, and electrochemical polishing. Subsequently, the Zr-4 sheets were rinsed with DI water and dried in an air stream at high pressure. The samples were anodized in EG (99.3 wt.%, extra pure; Junsei Chemical Co., Ltd., Tokyo, Japan) electrolyte containing 0.5 wt.% ammonium fluoride (NH_4_F, Sigma-Aldrich Corporation, St. Louis, MO, USA) and 0.2 wt.% DI water. The anodization process was carried out using a two-electrode system with platinum gauze (15 × 25 × 0.2 mm^3^) as a counter electrode and Zr-4 sheet as a working electrode. The distance between the cathode and anode was fixed at 10 mm. All the chemicals and materials were used in their as-received forms without any further purification. Anodization was conducted at a constant voltage of 40 V for 4 h at room temperature using a DC power supply. The anodization induces Zr-4 oxide nanotube arrays on the surface of Zr-4 sheets, and these nanotubes were washed with DI water many times and dried in air using high pressure. The as-anodized Zr-4 oxide nanotubes were annealed in air for 2 h at 450°C at the rate of 3°C/min. The structural morphology of the nanotubes was examined using a field emission scanning electron microscope (FESEM, Hitachi S-4800; Hitachi Ltd., Tokyo, Japan) and high-resolution transmission electron microscope (HRTEM, Tecnai G2 F30; FEI Company, Hillsboro, OR, USA). The cross-sectional FESEM images were taken from mechanically cracked samples. Elemental analysis was done using an energy dispersive X-ray (EDX) analyzer attached onto the HRTEM. The crystal structure and phase composition were identified with the help of glancing-angle X-ray diffractometer (GAXRD, D/MAX 2500 V, Rigaku Corporation, Tokyo, Japan) with Cu Kα radiation (*k* =1.5406 Å). X-ray photoelectron spectroscopy (XPS) analysis was carried out using a spectrometer (Sigma Probe, Thermo VG Scientific, Waltham, MA, USA) with an Al Kα excitation source operated at 15 kV and 7 mA. The binding energies of all elements were calibrated using the C1s peak at 284.0 eV.

## Results and discussion

**Figure 1:**
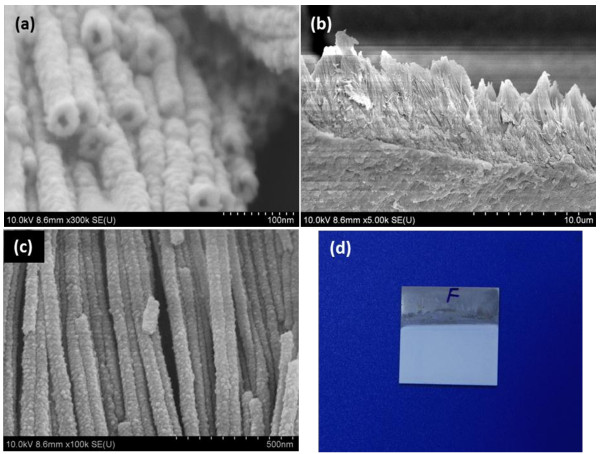
**FESEM images of the as-anodized Zr-4 oxide nanotubes.** The nanotubes were prepared in ethylene glycol (EG) electrolyte containing a small amount of NH_4_F and DI water showing the top **(a)** and cross-sectional **(b)**-**(c)** surface morphology. Digital photo of Zr-4 oxide nanotubes along with Zr-4 substrate **(d)**.

**Figure 2:**
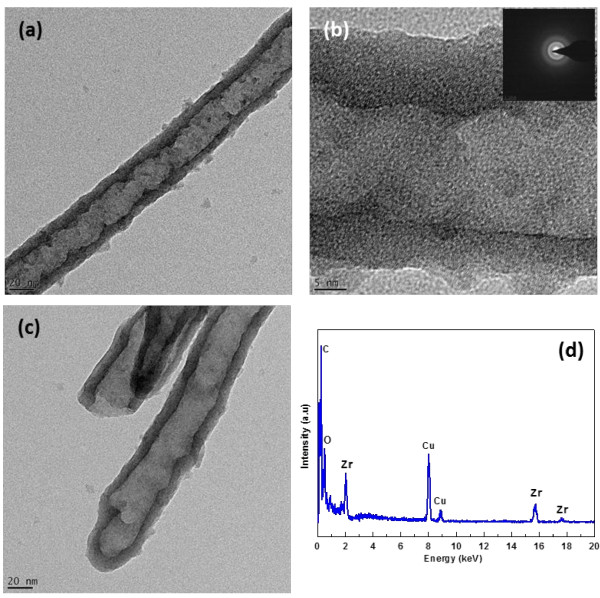
**TEM images and EDX spectrum of Zr-4 oxide nanotubes.** TEM images **(a)**-**(c)** showing the formation of Zr-4 oxide nanotubes in EG electrolyte containing a small amount of NH_4_F and DI water. EDX spectrum of Zr-4 oxide nanotubes **(d)**.

**Figure 3:**
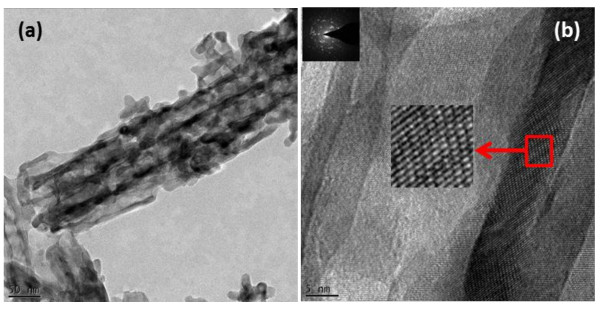
**TEM images of annealed Zr-4 oxide nanotubes.** The disintegration of tubular morphology **(a)** and increase in crystallinity **(b)** after annealing are shown.

**Figure 4:**
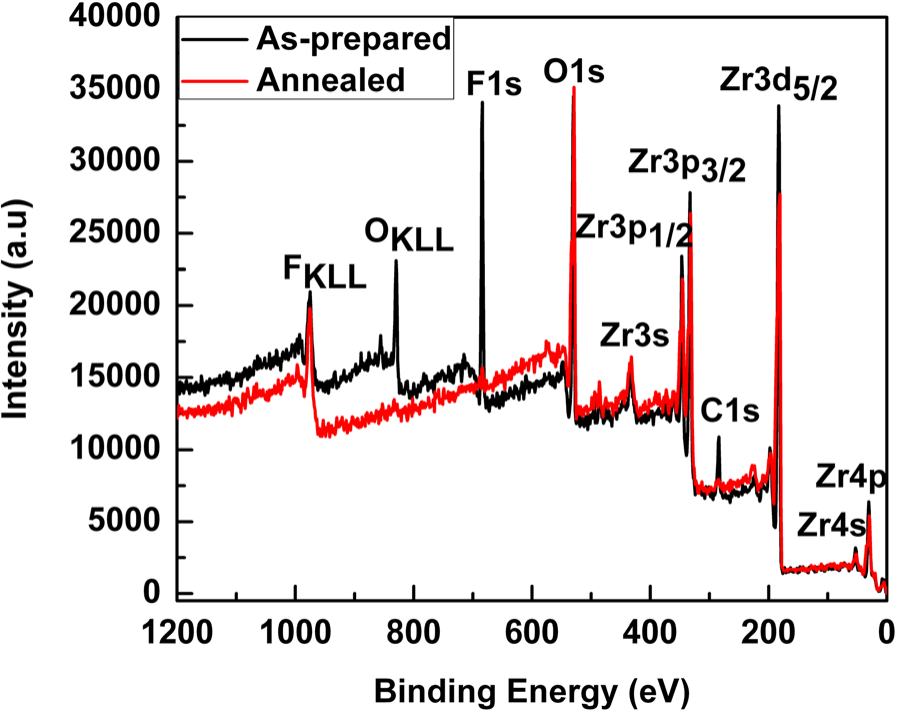
Wide-scan XPS spectra of the as-anodized and annealed Zr-4 oxide nanotubes.

**Figure 5:**
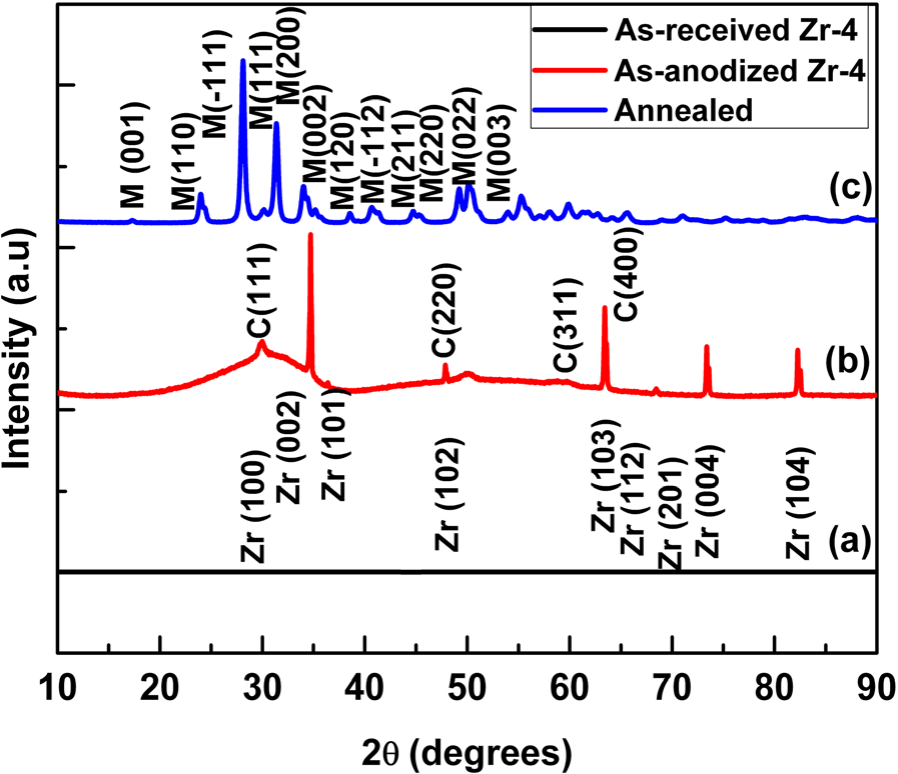
**XRD patterns.** As-received Zr-4 sheet **(a)**. As-anodized **(b)** and annealed **(c)** Zr-4 oxide nanotubes. XRD patterns of the Zr-4 oxide nanotubes clearly show that the as-anodized nanotubes were crystalline in cubic phase which is transformed into monoclinic after annealing.

**Figure 6:**
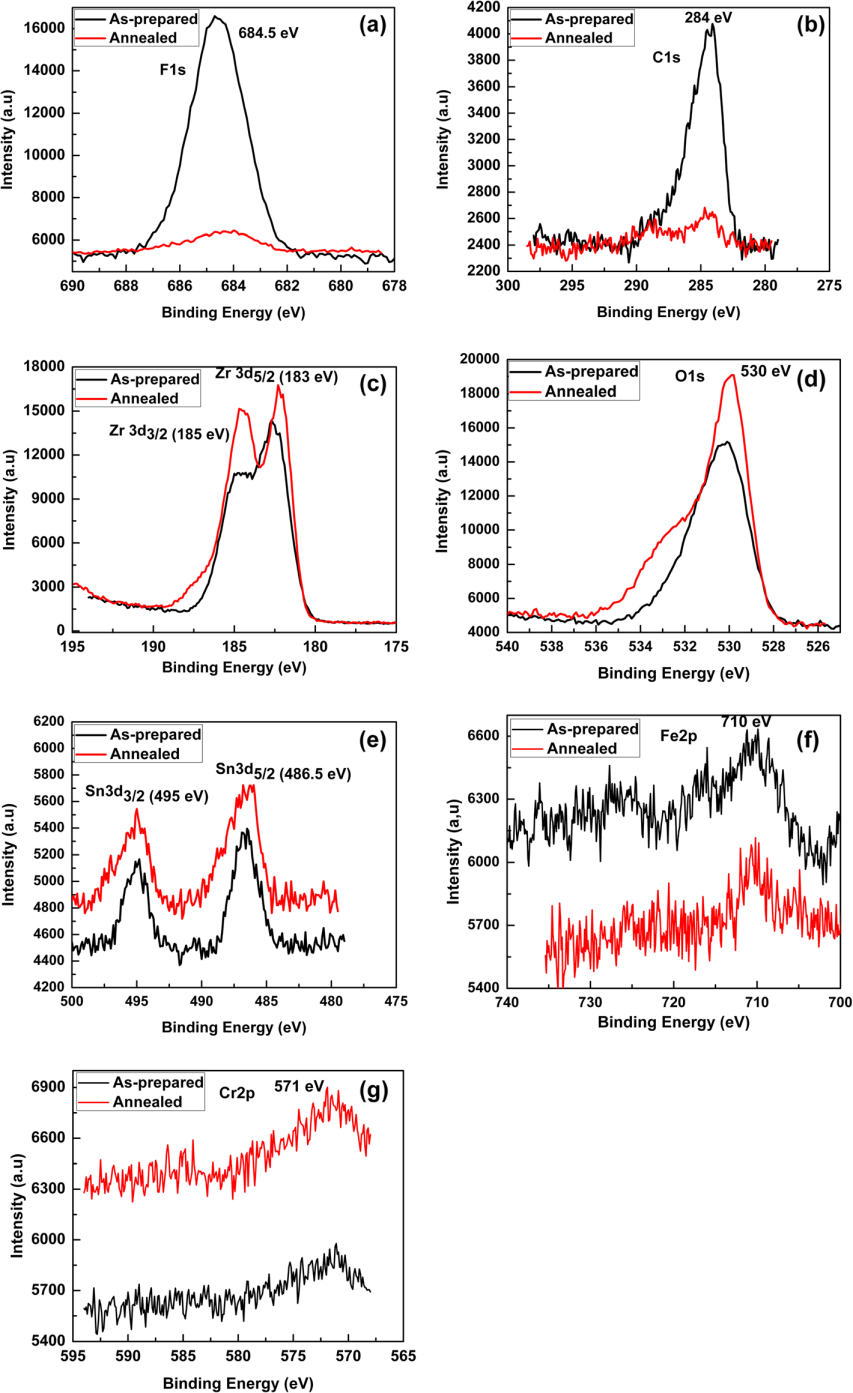
**High-resolution XPS spectra. (a)** F, **(b)** C, **(c)** Zr, **(d)** O, **(e)** Sn, **(f)** Fe, and **(g)** Cr of the as-anodized and annealed Zr-4 oxide nanotubes.

FESEM images of the as-anodized Zr-4 oxide nanotubes prepared in EG-based electrolyte are shown in Figure 1a,b,c. The top surface image (Figure 1a) confirms the formation of self-organized Zr-4 oxide nanotube. The exact diameter and wall thickness of Zr-4 oxide nanotubes cannot be determined from the SEM image due to the thick Pt coating layer. The length of the Zr-4 oxide nanotubes is approximately 13 μm as depicted in the cross-sectional image (Figure 1b). The high-magnification cross-sectional image (Figure 1c) reveals the straight morphology of the nanotubes along with thick Pt coating layer. Figure 1d shows the digital picture of the as-anodized Zr-4 oxide nanotubes. A compact morphology with good adhesion of the oxide layer with Zr-4 substrate can be seen in the digital photo. Figure 2 is the TEM images of the as-anodized Zr-4 oxide nanotubes along with EDX spectrum. The TEM image of the single Zr-4 oxide nanotube (Figure 2a) shows that the inner diameter of the nanotube is 20 nm with average wall thickness of approximately 7 nm. Moreover, it also reveals that the wall morphology of the nanotube is not very smooth like TiO_2_ nanotubes [10,11]. A small variation in the wall thickness of Zr-4 oxide nanotube at the same location can be found in the high-resolution TEM image (Figure 2b). This may be attributed to the difference in the dissolution rate of the different elements in the Zr-4 alloy due to F ions. The HRTEM image and SAED pattern (inset of Figure 2b) demonstrate that the as-anodized Zr-4 nanotubes are partially crystalline like TiO_2_ nanotubes [28]. Zr-4 oxide nanotube is closed at the bottom end (Figure 2c); however, the bottom wall morphology is also not very smooth and round like TiO_2_ nanotubes [10,11]. The EDX spectrum of the as-anodized Zr-4 oxide nanotubes (Figure 2d) generally shows the peaks of major elements like Zr and O along with C and Cu which resulted from the TEM grid. The morphology of Zr-4 oxide nanotubes after annealing was also examined by TEM, and the results are displayed in Figure 3. It can be seen from the TEM images that the tubular morphology and integrity of the Zr-4 oxide nanotubes shown in Figure 2 have deteriorated after annealing. This is mainly due the elimination of F species from Zr-4 oxide nanotubes after thermal annealing process [29]. Our XPS results (Figure 4) clearly demonstrate this fact. A complete change in the morphology from ZrO_2_ nanotubes to nodular nanostructure was reported by Lee et al. [29] after the elimination of the F species from anodic ZrO_2_ nanotubes by heat treatment. Similarly, the removal of F species from anodic TiO_2_ nanotubes leads to the formation of truncated [30,31] and concatenated TiO_2_ nanoparticles after annealing process [32,33]. F species usually remained trapped in the anodic metal oxide nanotubes layer during anodization in F-containing electrolytes which can be easily removed by heat treatment [29,32-35]. Figure 3b shows the HRTEM of a single Zr-4 oxide nanotube along with SAED pattern (the inset image). It indicates that the crystallinity of the Zr-4 oxide nanotubes was enhanced after annealing. Figure 5 shows XRD patterns of the as-received Zr-4 sheet as-anodized and annealed Zr-4 oxide nanotubes. All peaks in the XRD patterns are indexed and labeled according to their crystalline planes. It can be seen that XRD pattern of the as-received Zr-4 (Figure 5a) mostly gives peaks of zirconium metal (JCPDS card no. 05-0665) which is consistent with literature [6]. The XRD pattern of the as-anodized sample (Figure 5b) clearly reveals the formation of crystalline cubic phase (JCPDS card no. 05-0665). It is reported that the cubic phase predominantly appeared in the as-anodized zirconium metal [17,18,22] and Zr-2 [25]. The presence of Sn in zircaloy usually stabilized the cubic ZrO_2_ phase [25]. The presence of Sn in the Zr-4 oxide nanotubes has been confirmed by the XPS results (Figure 6e). The cubic phase is mostly transformed into baddeleyite monoclinic phase (JCPDS card no. 37-1484) after annealing; however, few peaks of the cubic phase can be also found in the XRD spectrum (Figure 5c). The XRD results indicate that the annealing process enhanced the crystallinity of the Zr-4 oxide nanotubes. Thus, on the one hand, the annealing process enhanced the crystallinity of the Zr-4 oxide nanotubes while on the other hand, it disintegrates the tubular morphology. The chemical compositions of the as-anodized as well as the annealed Zr-4 oxide nanotubes were determined using XPS. The XPS results of the as-anodized and the annealed Zr-4 oxide nanotubes are shown in Figures 4 and 6. Figure 4 shows the wide-scan (survey) spectra, while Figure 6a,b,c,d,e,f,g shows the high-resolution spectra of the Zr-4 oxide nanotubes. The wide-scan spectra of the as-anodized as well as the annealed Zr-4 oxide nanotubes (Figure 4) reveal the dominant peaks of Zr and O, which confirm the formation of Zr-4 oxide. F and C peaks can also be seen in the survey spectra; however their intensities were tremendously reduced after annealing process as shown in the high resolution spectra (Figure 6a,b). It is known that anodization of metals in F-containing electrolytes always resulted of F- trapping in the oxide layer as F ions are in competition with O ions during the growth of metal oxide layer in anodization [32,34]. This has been confirmed from our XPS results (Figure 6a) of the as-prepared Zr-4 oxide nanotubes, which shows that a huge amount of F species has been incorporated in the Zr-4 oxide nanotubes during anodization; however, the amount of F was greatly decreased after the annealing process (Figure 6a). The peak located at 684.5 eV in the high-resolution spectra of F1s (Figure 6a) can be assigned to the Zr-4 oxide-fluoride-type compound [36]. The presence of C species (C1s peak) in the as-prepared Zr-4 oxide nanotubes (Figure 6b) is due to the use of organic electrolyte for anodization of Zr-4. The intensity of C peak was greatly reduced after annealing process (Figure 6b). The high-resolution spectra of Zr before and after annealing are shown in Figure 6c. It shows two dominant peaks located at 183 and 185 eV. The XPS peaks located at 183 and 185 eV can be assigned to Zr3d_5/2_ and Zr3d_3/2_ respectively, exhibiting the fully oxidized state of Zr^4+^. These results are in good agreement with the reported literature [21,37]. The high-resolution spectra of oxygen in the as-prepared and the annealed Zr-4 oxide nanotubes are depicted in Figure 6d. It shows a single dominant peak located at 530.0 eV which can be attributed to the lattice oxygen of ZrO_2_. In addition, a broad shoulder peak in the energy range of 533 eV can be seen in the annealed Zr-4 oxide nanotubes. This peak can be attributed to the presence of hydroxyl species bond [35,37]. Figure 6e gives the high-resolution XPS spectra of Sn in the as-anodized and the annealed Zr-4 oxide nanotubes. The spectra clearly show two prominent peaks located at 486.5 and 495 eV which can be assigned to Sn3d_5/2_ and Sn3d_3/2_ respectively [38,39]. The high-resolution XPS spectra of Fe and Cr are shown in Figure 6f,g, respectively. The XPS spectra of these elements show broad peaks, demonstrating that these elements are present in the Zr-4 alloy in trace amount. The XPS peak of each element (Fe and Cr) is marked in the respective spectra which correspond to the oxide state of the respective elements [36]. The intensities of these peaks are very low due to their low concentration of these elements in the Zr-4.

## Conclusions

In summary, self-organized Zr-4 oxide nanotubes were fabricated on Zr-4 sheet in EG electrolyte containing a small amount of NH_4_F and DI water via anodization at room temperature. SEM and TEM results showed that the length of Zr-4 oxide nanotubes is approximately 13 μm; the inner diameter is approximately 20 nm with wall average thickness of approximately 7 nm. The wall morphology of Zr-4 oxide nanotube is not very smooth and homogeneous. The XRD results confirm the formation of a single cubic crystalline phase in the as-anodized form and co-existence of cubic as well as monoclinic phases after annealing. The structural morphology of the Zr-4 oxide nanotubes was disintegrated after annealing due to the elimination of F species.

## Competing interests

The authors declare that they have no competing interests.

## Authors’ contributions

GA conceived the original idea, carried out most of the experiments, and drafted the manuscript. YJP helped in the anodization experiments. HJK arranged the materials and chemicals and carried out the morphology characterization. SOC supervised the research process and provided constructive opinions to improve the quality of the research. All authors read and approved the final manuscript.
